# How does ethnicity affect presence of advance care planning in care records for individuals with advanced disease? A mixed-methods systematic review

**DOI:** 10.1186/s12904-023-01168-7

**Published:** 2023-04-17

**Authors:** Jodie Crooks, Sophie Trotter, Patient Public Involvement Consortium, Ruby Bhatti OBE, Elizabeth Monaghan, Gemma Clarke

**Affiliations:** 1 Research and Policy, Marie Curie, London, UK; 2 Academic Unit of Palliative Care, University of Leeds, Leeds, UK

**Keywords:** Ethnicity, Race, Advance care planning, Health inequalities, Advanced disease, Palliative care, Systematic review

## Abstract

**Background:**

Advance care planning (ACP) is the process supporting individuals with life-limiting illness to make informed decisions about their future healthcare. Ethnic disparities in ACP have been widely highlighted, but interpretation is challenging due to methodological heterogeneity. This review aims to examine differences in the presence of documented ACP in individuals’ care records for people with advanced disease by ethnic group, and identify patient and clinician related factors contributing to this.

**Methods:**

Mixed-methods systematic review. Keyword searches on six electronic databases were conducted (01/2000–04/2022). The primary outcome measure was statistically significant differences in the presence of ACP in patients’ care records by ethnicity: quantitative data was summarised and tabulated. The secondary outcome measures were patient and clinician-based factors affecting ACP. Data was analysed qualitatively through thematic analysis; themes were developed and presented in a narrative synthesis. Feedback on themes was gained from Patient and Public Involvement (PPI) representatives. Study quality was assessed through Joanna Briggs Institute Critical Appraisal tools and Gough’s Weight of Evidence.

**Results:**

N=35 papers were included in total; all had Medium/High Weight of Evidence. Fifteen papers (comparing two or more ethnic groups) addressed the primary outcome measure. Twelve of the fifteen papers reported White patients had statistically higher rates of formally documented ACP in their care records than patients from other ethnic groups. There were no significant differences in the presence of informal ACP between ethnic groups. Nineteen papers addressed the secondary outcome measure; thirteen discussed patient-based factors impacting ACP presence with four key themes: poor awareness and understanding of ACP; financial constraints; faith and religion; and family involvement. Eight papers discussed clinician-based factors with three key themes: poor clinician confidence around cultural values and ideals; exacerbation of institutional constraints; and pre-conceived ideas of patients’ wishes.

**Conclusions:**

This review found differences in the presence of legal ACP across ethnic groups despite similar presence of informal end of life conversations. Factors including low clinician confidence to deliver culturally sensitive, individualised conversations around ACP, and patients reasons for not wishing to engage in ACP (including, faith, religion or family preferences) may begin to explain some documented differences.

**Trial registration:**

PROSPERO-CRD42022315252.

**Supplementary Information:**

The online version contains supplementary material available at 10.1186/s12904-023-01168-7.

## Background

### Rationale

Advance Care Planning (ACP) is an internationally recognised term describing conversations around an individual’s future healthcare preferences. The aim is to support individuals to “live well and die well, in the place of their choosing” [1]. These conversations can lead to legal outcomes including (but not limited to) lasting or durable power of attorney, advanced directives/statements, and living wills (“a decision you can make now to refuse a specific type of treatment at some time in the future”) [2, 3]. ACP’s acceptability to patients is variable for many reasons, including the challenge of having to consider a deterioration in health; preference to spend their remaining time with family and friends; and other practical difficulties such as language barriers and health literacy [2].

The first national guidance for healthcare professionals in the UK was developed in 2007 [4]. Since then, ACP has become a fundamental component of patient-centred care. In the US, ACP development has a longer history [5] with legal tools of ACP evolving from the mid-1970s [6]. Notably, ACP is an internationally recognised term used by healthcare systems in over 40 countries [1]. In 2017, an international taskforce composed a Delphi study where over 100 experts in the field (across Europe, North America and Australia) collaborated to build an international consensus and create practice recommendations for ACP [7]. The resulting consensus defined: “Advance care planning enables individuals to define goals and preferences for future medical treatment and care, to discuss these goals and preferences with family and healthcare providers, and to record and review these preferences if appropriate”. This was the first transcultural consensus definition of ACP, aiming to unify approaches internationally.

### Race, ethnicity & ACP

The international literature on race, ethnicity and ACP is complex due to variations in the definition and measurement of ACP, the wide range of disease groups and differences in labels for ethnic groups used across different countries. In this study, ACP is defined as “a process that supports adults at any age or stage of health in understanding and sharing their personal values, life goals, and preferences regarding future medical care” [8]. The current review focusses on ethnicity, defined by the American Psychological Association’s (APA) guidance for reporting ethnicity as “referring to shared cultural characteristics such as language, ancestry, practices and beliefs” [9]. Although the current review will not refer to groups by ‘race’, some individual studies considered within the review utilise the term. Therefore, the following definition of race is offered by the APA: ““race refers to physical differences that groups and cultures consider socially significant”.

There are long recognised ethnic inequalities in healthcare [10, 11]. Over recent years there has been an increased focus on these disparities, catalysed by factors including the Black Lives Matter movement [12] and the unequal number of deaths during the Covid-19 pandemic [13, 14]. Many studies internationally indicate some degree of disparity in rates of ACP related to ethnicity [15-17]. Evidence from the USA suggests that White patients have higher rates of hospice use, and formal ACP documentation and/or “do not attempt cardiopulmonary resuscitation” (DNACPR) statements compared with people from other ethnic groups [18-20]. Specifically, White patients are evidenced to be two to three-times more likely to have an advanced directive than Black or Latino patients [21]. This disparity also appears to be consistent across other countries; UK-based data suggests White patients are more likely to have DNACPR statements orders than any other patient group [22]. The issues underlying these disparities are complex and likely multifactorial; some ACP literature notes specific differences in ethnic values related to illness, dying and decision making, as well as differences in disease and comorbidity burden [23], education levels [24], and family involvement [25]. Furthermore, broader considerations include systemic racism and power imbalances, described as a legacy of colonial oppression that remains felt within today’s society and healthcare system [26]. These issues intersect with ethnicity to cause inequity in ACP [27].

### Previous systematic reviews

A review of reviews [28] highlighted the heterogeneity of research exploring the role of ethnicity on ACP and emphasised the importance of starting to consolidate this. Previous reviews, for example, include those synthesising disparities in the presence of ACP by ethnicity, reviews investigating the underlying reasons for this, and reviews identifying ethnicity as a factor affecting ACP [29-31].

Bazargan & Bazargan-Hejazi (2021)’s review investigated disparities in ACP document completion for non-Hispanic Black patients [29]. They found “major differences” in the general rate of advanced directives completed: patients from non-Hispanic White groups had higher completion rates than patients from non-Hispanic Black groups and all other ethnic groups assessed. This finding remained when specifically studying individuals with advanced disease; non-Hispanic White patients with a serious illness were more likely than non-Hispanic Black patients to have legal ACP documentation.

Other reviews identify ethnicity as a factor influencing presence of ACP, though the underlying reasons for this require further exploration. For example, Lovell and Yates (2014) found that “being African American” affected uptake of ACP in palliative care [30], and Spelten et al. (2019) found that White patients were more likely to be involved in ACP than Latino or African American patients [31]. Some reviews have investigated these factors in greater depth; for example, McDermott & Selman (2018) found specific cultural factors affecting ACP in patients with advanced disease included religiosity, trust in the healthcare system and patient and clinician comfort discussing death [32]. Similarly, Hong et al. (2018) found health literacy and experiences, cultural values, and spirituality to be factors affecting ACP presence [33]. Notably, Sanders et al. (2016) utilised a systematic review methodology to produce a model beginning to explain factors impacting ACP presence among African American patients [34]; this identified patient-based factors and system-specific factors impacting ACP presence, including trust, family, beliefs about illness and death, and religion and spirituality. However, this review only considered African American patients.

The heterogeneity of research studies (and to some extent, reviews) elicits the need for an up-to-date review encapsulating global literature on ACP for all ethnically diverse groups. This review aims to investigate the existing literature around ethnic disparities in the documented presence of ACP and to illustrate the patient and clinician factors affecting this, to improve understanding of culturally competent ACP and promote progression towards this.

### Research objectives

This study will review published literature around ACP and ethnicity. Firstly, we will explore and illustrate disparities in the documented presence of ACP by ethnicity; secondly, we will conduct thematic analysis of extracted data to illustrate key clinician and patient-based factors affecting this.

## Methods

### Study design

This is a mixed-methods systematic literature review including (1) tabulation and narrative exploration of the primary outcome measure (i.e., differences in presence of ACP by ethnicity) and (2) a Thematic Analysis [35] of qualitative data illustrating the secondary outcome measure (i.e., factors related to ethnicity affecting the presence of ACP). The quantitative and qualitative analyses are conducted and presented separately, allowing us to address each research objective, and then considered in combination within the discussion section of the review: a parallel-results convergent design [36, 37]. The current study was prospectively registered on PROSPERO (registration number: CRD42022315252) and was undertaken in accordance with PRISMA 2020 guidelines (Additional 1) [38].

### Eligibility criteria

The full eligibility criteria are outlined in Table 1 below. Although ACP can be useful for healthy adults, this review focussed on adults with advanced disease. This was due to the perceived high importance of ACP for those with an advanced, life-limiting condition within the literature, and the quantity of research publications within the field. Criteria relating to advanced disease were purposefully broad and inclusive to maximise transferability; we included papers involving patients with any advanced, incurable, life-limiting condition or disease. Notably, we excluded populations where advanced disease was not explicitly reported (i.e. ‘older adults’ or ‘nursing home residents’), as this review aimed to focus on ACP for those with advanced disease only. Research published between 2022 and 2000 was searched; the year 2000 was chosen as the lower limit to allow inclusion of a broad range of literature, while acknowledging that ACP practices have developed over time.

**Table 1:** Eligibility criteria for including papers in review

	**Inclusion Criteria**	**Exclusion Criteria**
Population	• Patients with any advanced, incurable, life-span limiting condition or disease - Can include multi-morbidity (so long as at least one condition is advanced) - Can include ‘serious illnesses’ that have led to palliative care consultation • Proxy discussions of any ‘real patients’ (e.g., by a relative/carer or healthcare professional) where the patient otherwise meets the inclusion criteria • (For RQ2) Any healthcare professional (e.g., consultant, doctor, nursing staff) • Any self-defined ethnicity • Adults > = 18-years-old	• Patients with non-advanced/non-lifespan-limiting conditions only • The above includes ‘nursing home’ and ‘community dwelling older adult’ populations where no advanced disease is reported • Patients with curable disease • Children/paediatric patients under 18-years-old • Members of the general public with no advanced disease
Comparison	• Either: - Contains a comparison between two or more groups based on ethnicity/race - RQ2: If focussing on a single group, contains a substantive focus or analysis of cultural, ethnic or racial factors	• Focus on only a single group with no analysis of cultural/ethnic factors
Outcome Measures	• RQ1: Any exploration/discussion of uptake of advance care planning (i.e., qualitative, or quantitative) • RQ2: Any exploration/discussion of patient or clinician factors affecting advance care plan uptake (likely qualitative but include quantitative)	• No exploration or discussion of either: - Uptake of advance care planning - Factors affecting uptake of advance care planning
Study Design & Setting	• Any qualitative or quantitative study design (including case reports where ethnicity is discussed) • Any location of healthcare (including but not limited to hospice, hospital, community, home care) • Year of publication 2000 – 2022 • English language publication • Data from any country	• Papers with no new empirical data or new analyses (including systematic reviews, editorials, and commentaries) • Case reports with no reference to ethnicity • Published before year 2000 • Non-English language publication • Autopsy, wet-lab and animal studies

### Information sources

Searches were conducted on six key databases on 16/03/2022: PubMed (2000 – March 2022; Medline (2000 – March 2022); AMED (2000 – March 2022); EMBASE (2000 – March 2022); PsychINFO (2002 – present); CINAHL (2000 – present). Additional literature was sought via manual journal index searching on 06/04/22 (using criteria 2000 – 06/04/22) of: Palliative Medicine; BMJ Supportive and Palliative Care; BMC Palliative Care; Ethnicity and Healthcare.

### Selection and data collection processes

Search outputs were imported into a reference managing software (EndNote) and records deduplicated. Two members of the research team (JC, ST) independently screened the remaining titles for inclusion/exclusion; decisions were reviewed (JC, ST) and discrepancies resolved through discussion (JC, ST, GC). Abstracts and subsequently full texts were selected through the same process.

Data extraction of full texts was conducted independently by two members of the research team (JC, ST) into a pre-designed form, extracting study data, cohort data and data relating to both the primary and secondary outcome measures. Output was compared and combined. Discrepancies were resolved through discussion with a third team member (GC).

### Outcome measures

#### Primary outcome measure

The primary outcome measure was statistically significant differences in the documented presence of advance care planning in individuals’ care records for people with advanced disease by ethnicity; this is illustrated in the tabulation (see Table. 4). For the primary outcome analysis, only papers comparing two or more ethnic groups as defined by the original paper were included in order to facilitate measuring differences. Studies not reporting data on the primary outcome measure were excluded from the primary outcome analysis. Due to the heterogeneous nature of ACP within research literature, we included a variety of measurement tools for ACP including current and retrospective care records, formal documentation of ACP (including advance directives, DNACPR, living wills), patient/carer self-reports, and any other in-study designed measurement of ACP occurrence. Cross-sectional, retrospective, and longitudinal studies were all included. For longitudinal studies, advanced disease must have been present at study commencement; data collected throughout the study was included in this analysis.

### Secondary outcome measure

The secondary outcome measure was clinician and patient-based factors affecting the presence of advanced care planning. This was extracted from papers analysing a single ethnic group with substantive focus on cultural or ethnic factors; and from papers comparing two or more ethnic groups (as defined by the paper) to enable a comprehensive exploration. This was a qualitative analysis considering factors in two key categories: patient-related factors and clinician-related factors.

Other data extracted included study data (i.e., geographic location, healthcare location, main objective) and cohort data (participant type, participant demographics including age and gender, ethnic groups discussed, disease type).

### Risk of bias

Risk of bias assessment was conducted using the Joanna Briggs Institute Critical Appraisal Tools [39]. The appropriate critical appraisal tool was selected according to the paper’s methodological design and completed independently by two researchers (JC, ST) for each accepted full text. These weightings were used to inform the tabling and narrative synthesis analyses, and interpretation of results: papers were scored as high (90–100%), medium (80–90%) or low (70–80%); those scoring less than 70% were excluded.

To consider the suitability of each included paper, Gough’s (2007) Weight of Evidence (WoE) assessment was conducted (JC, ST) with the JBI outcome integrated into this as WoE criteria A [40]. The other two components of WoE (i.e., the appropriateness of research methodology and the relevance of research question and outcome) were assessed independently by two members of the research team (JC, ST). All three components were combined to produce one overall WoE assessment (high, medium, or low), used as both a sensitivity analysis and certainty analysis to assess robustness and confidence in synthesised results, respectively. Risk of Bias and Weight of Evidence ratings can be found in the Study Characteristics table (Table 3).

### Synthesis methods & analysis plan

To decide which studies were eligible for each synthesis, study characteristics were tabulated (Table 3) and compared against the planned outcome measure for each synthesis.

Data extracted for the primary outcome measure (statistically significant differences in the presence of documented ACP in individuals’ care records for people with advanced disease by ethnic group) was quantitatively tabulated to demonstrate the key findings. To represent confidence in the results and compare results of similar quality papers, output was organised by Weight of Evidence ratings. No numerical data conversions occurred. Inter-related ethnic groups were tabulated together for the summary analysis (Table 2) for concision. These summary groupings were reviewed for appropriateness by Patient and Public Involvement contributors who were ethnically diverse individuals with experience (personal or via a loved one) of ACP and advanced disease. No meta-analysis was conducted due to the heterogeneity of ethnic groupings and ACP measurement tools within the literature.

**Table 2:** Ethnic groupings used for summary analysis

Original racial and ethnic group names used in papers	Summary analysis group name	Number of papers
White	White	24
Caucasian		
Non-Hispanic White/Caucasian		
Non-Latino White New Zealand European^a^ Other European^a^		
Black	Black & African American	27
African American		
Non-Hispanic Black		
Asian	Asian	9
Chinese	(South) East Asian	7
Taiwanese		
Malay		
Latino/a	Hispanic	11
Hispanic White		
Māori	Māori	1
Non-Māori	Non- Māori	1
Pacific	Pacific	3
Non-Pacific	Non-Pacific	1
Mixed-race (i.e., Chinese American, Asian American) Native [American, Indian, Hawaiian]	Minority Other	5

^a^The terms ‘New Zealand European’ and ‘Other European’ were used in reference [41]. The authors understand these terms to be in reference to ‘White’ ethnic groups

For the secondary outcome measures (clinician and patient -based factors affecting presence of ACP for different ethnic groups), extracted data was imported into a qualitative coding software (NVivo Plus™). Analysis followed thematic analysis methodology as described by Braun & Clarke [35]. Initial coding was undertaken independently by two researchers (JC, ST) who then collaborated for reiterative coding and theme development. Themes were explored within two main categories: patient-related and clinician-related factors affecting presence of ACP. Within these two categories, coding and theme development was inductive. Initial themes were refined through discussion with the wider research team (GC) and through Patient and Public Involvement.patient & public involvement

Two Patient and Public Involvement (PPI) contributors provided feedback to inform this review: a British-South Asian individual with personal and professional experience of ACP, and a White Other, Jewish individual with personal experience of ACP. Early iterations of themes illustrating the secondary outcome measure were presented to the PPI contributors, who offered comments and opinions based on their own personal and professional experiences. This feedback and critique shaped the final iterations of the themes presented. PPI contributors were also invited to be co-authors for this review, and were involved in reviewing and giving feedback for drafts of the review manuscript.

## Results

### Study selection

**Fig. 1:**
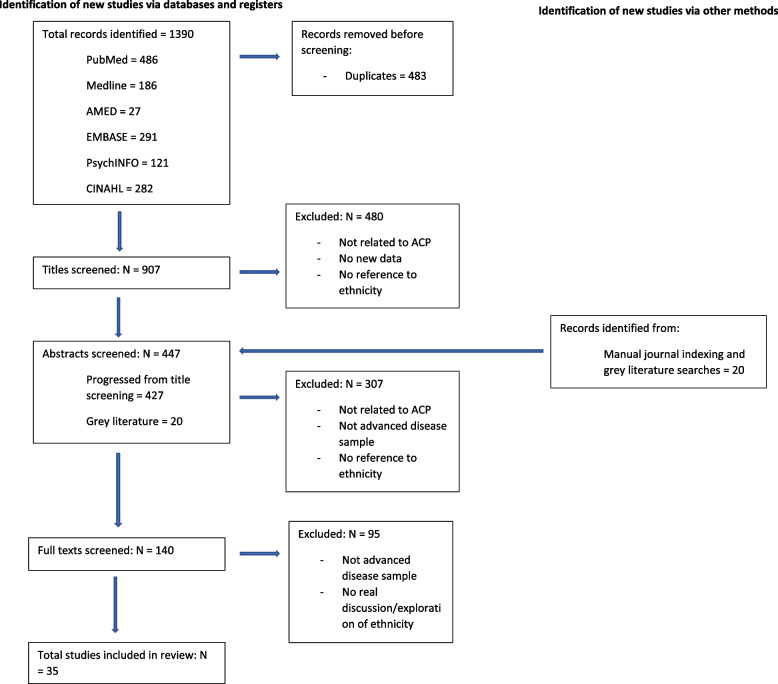
PRISMA diagram of studies excluded at each stage of screening

The electronic searches yielded 1390 papers of which 907 remained eligible after de-duplication. The screening process diagrammed in Fig. 1 resulted in 35 papers being included in the review.

### Study characteristics

The characteristics of the 35 studies included in the analysis, along with the Risk of Bias and Weight of Evidence ratings, are presented in Table 3.

**Table 3:** Characteristics of included studies

First Author Year Country	Study Population (n)	Ethnic Group(s)^a^ (%)	Disease Group/Clinician Type	Healthcare Location	Summary objectives and methods	JBI Critical Appraisal Score^b^	Gough WoE^c,d^	Primary measure	Secondary measure
Beltran 2022 USA [42]	Patients (13) Relatives (10)	Latino (100%)	Cancer Heart disease Dementia COPD ALS Renal failure	Hospice	Qualitative semi-structured interviews to explore factors affecting Latino patients’ decisions to enrol in hospice care	85%	H		Y
Braun 2010 USA [43]	HCPs (26)	Caucasian (42.3%) African American (30.7%) Hispanic (26.9%)	Generalists Sub-specialists	Medical centre	Focus group interviews to explore physician factors affecting end of life decision making	89%	H		Y
Eneanya 2016 USA [44]	Patients (152)	Black (41.4%) White (58.6%)	End-stage kidney disease	Academic centres	Qualitative semi-structured interviews to explore racial variability in ACP	100%	H	Y	
Li 2021 Taiwan [45]	Patients (9)	Taiwanese /Indigenous (100%)	Cancer	Not reported	Case study approach to explore indigenous patients perceptions of ACP	90%	H		Y
Lin 2020 Taiwan [46]	Patients (10) Carers (10) HCP (9)	Taiwanese (100%)	Cancer	Inpatient hospital	Mixed methods study (qualitative interview and quantitative review of medical records) to examine feasibility of culturally-sensitive ACP intervention	95%	H		Y
Mack 2010 USA [47]	Patients (332)	Black (21.4%) White (78.6%)	Cancer	Varied cancer services/clinics	Longitudinal study to investigate end of life care disparities between “Black and White” patients	90%	H	Y	
Periyakoil 2015 USA [48]	HCP (1040)	Caucasian (48.7%) Latino-American (5.8%) African American (3.4%) Asian (33.6%) Mixed race & ethnicity (8.4%)	Multi-speciality doctors	Academic medical centre	Cross-sectional survey to identify clinician barriers to end of life conversations with ethnically diverse patients	94%	H		Y
Pettigrew 2020 USA [49]	Patients & carers (431)	Carer: White (87.2%) African American (9.8%) Other (3%) Patient: White (88.2%); African American (10.2%); Other (1.6%)	Dementia	Dementia clinics	Cross-sectional survey to understand factors affecting ACP decision making in persons with dementia	92.5%	H	Y	
Rhodes 2015 USA [50]	HCPs (12)	Non-Hispanic White (83.3%) Non-Hispanic Black (8.3%) Other (8.3%)	Physician Nursing staff Social workers Chaplains	Hospice	Qualitative semi-structured interview to explore barriers to use of end of life care options with HCPs	82.5%	H		Y
Shen 2016 USA [51]	Patients (117)	Latino (52.1%) Non-Latino/White (47.9%)	Cancer	Public hospital	Structured interviews to explore ethnic differences in the association between end of life discussions and DNR completion	96.5%	H	Y	
Ashana 2022 USA [52]	HCPs (74)	White (92.4%) Asian (7.6%) Black (1.5%)	Physicians Nurses Social workers Chaplains	Varied healthcare centres	Qualitative semi-structured interviews to identify populations less approached for ACP, in addition to clinician-level barriers and facilitators	77.5%	M		Y
Burgio 2016 USA [53]	Patients (4891)	African American (34.5%) White (65.3%)	Cancer Dementia Heart disease Lung disease Kidney disease Liver disease	VA Medical centre	Retrospective review of medical records to explore differences between African American and White patients	82.5%	M	Y	
Cervantes 2017 USA [54]	Patients (20)	Latino (100%)	End-stage kidney disease	Outpatient haemodialysis centre	Semi-structured qualitative interviews to understand ACP preferences of Latino patients	80%	M		Y
Cheung 2020 Hong Kong [55]	Patients (17) Relatives (13)	Chinese (100%)	Not reported	Palliative day care centre	Qualitative interviews and focus groups to explore barriers to ACP	75%	M		Y
Frey 2014 New Zealand [41]	HCP (40)	New Zealand European (47.5%) Maori (7.5%) Pacific (7.5%) Asian (12.5%) Southeast Asian (17.5%) Other European (7.5%)	Staff nurse GP Anaesthetist Consultant	Palliative care services	Interviews and focus groups to explore HCP perspectives on the barriers to ACP	75.5%	M		Y
Garrido 2014 USA [56]	Patients (606)	Non-Latino White (72.1%) Black (15.3%) Latino (12.5%)	Cancer	Outpatient clinics	Baseline interviews from a longitudinal study to identify intervention targets for reducing disparities in ACP	75%	M	Y	
Grill 2021 USA [57]	Patients (223)	African American (86.1%) Non-African-American (11.2%)	HIV	Hospital-based clinics	Cross-sectional survey to explore factors affecting HIV patients end of life preferences	81.25%	M	Y	
Jonnalagadda 2012 USA [58]	Patients (335)	Non-minority (59.1%) Black (20.8%) Hispanic (20%)	Lung cancer	Medical centres	Cross-sectional survey to evaluate differences between “minority and non-minority” patients with lung cancer	80.75%	M		Y
Kirtane 2018 USA [59]	Patients (9468)	White (84.2%) Black (4.8%) Native American (1.2%) Asian (6.4%) Pacific Islander (0.5%) Hispanic (1.7%) Other/mixed race (1.1%)	Cancer	Medical centres	Retrospective cohort study and chart review to explore end of life outcome measures in “ethnic minority patients”	88.75%	M	Y	
Menon 2018 Singapore [60]	HCPs (33) Patients (15) Carers (13)	HCPs: Chinese (84%) Malay (3%) Indian (6%) Other (6%) Patients/Carers: Chinese (82.1%) Malay (7.1%) Indian (10.7%)	Doctors/nurses Not reported – ‘varied life limiting illnesses’	‘Varied healthcare centres’	Qualitative interviews and focus groups to study patient, relative and HCP perspectives on ACP	82.5%	M		Y
Periyakoil 2014 USA [61]	HCPs (1081) *[2013 sample]*	White/Caucasian (51.1%) Hispanic or Latino American (5.3%) African American (3.2%) Asian American (32.7%) Other (7.8%)	Doctors	(Training) Hospital	Online survey to determine clinician factors affecting advanced directives	83.125%	M		Y
Phipps 2003 USA [62]	Patients (68) Carers (68)	Patients: African American (55.8%) White (44.2%) Carers: African American (52.9%) White (47.1%)	Cancer	Cancer centre	Structured interview to explore end of life preferences in terminally ill patients	78.125%	M	Y	
Rhodes 2017 USA [63]	HCPs (14) Patients (1) Carers (2)	African American (100%)	Dementia Cancer	Hospice	Semi-structured interviews (and focus groups) to explore perceptions of ACP among African Americans	77.5%	M		Y
Sharma 2011 USA [64]	Patients (238)	White (63%) Black (32%) Other (5%)	Cancer	Hospital	Retrospective chart review to explore association between patient characteristics and ACP	87.25%	M	Y	
Shen 2020 USA [51]	Patients (20) Carers (9)	Patient: White/Latino (90%) Black (10%) Carer: White/Latino (66.7%) Other (33.3%)	Cancer	Outpatient cancer clinics	Qualitative interviews to explore patient and caregiver preferred ACP communication methods	80%	M		Y
Smith 2007 USA [65]	Patients (803)	African American (14.3%) White (85.7%)	Cancer Heart disease COPD Renal disease	Not reported	Cross-sectional survey to explore how the patient-physician relationship impacts ACP for African American and White patients	92.5%	M	Y	
Smith 2008 USA [15]	Patients (449)	White (69.4%) Black (16.7%) Hispanic (13.8%)	Cancer	Cancer clinics	Cross-sectional structured interview to explore proposed mediators of racial differences in ACP	80%	M	Y	
Taylor 2015 USA [66]	Patients (189)	White (60%) Hispanic (20%) Black (16%) Asian (4%)	Cancer	Cancer centre	Retrospective chart review to identify disparities in uptake of end of life resources	76%	M	Y	
Ufere 2019 USA [67]	HCP (396)	White (63.1%) Asian (29.5%) Black (3%) Native Indian/ Native Hawaiian (0.8%) Other (3.5%)	Hepatologists Gastroenterologists	Hospital / Private practice	Cross-sectional survey to explore HCP perspectives on barriers to ACP discussions	90%	M		Y
Zaide 2012 USA [68]	Patients (400)	White (47%) African American (35%) Asian (11%)	Cancer	Inpatient palliative care service	Retrospective chart review to explore how palliative care consultation impacts the rate of advanced directive completion	87%	M	Y	
Jung-Hwa 2021 Korea [69]	HCP (12)	Korean (58.3%) Korean-American (16.6%) White (16.6%) African American (8.3%)	Social workers	Hospital Day centres	Qualitative interviews to explore how social workers engage in ACP with a variety of patients	77.5%	M		Y
Chiu-Chu 2019 Taiwan [70]	HCP (221)	Taiwanese (91.7%) Mainlander (3.7%) Hakka (3.2%) Aborigine (1.4%)	Nurses	Teaching hospital	Structured questionnaire to explore ACP within a hospice environment in Taiwan	86.5%	M		Y
Wilkinson 2016 UK [71]	Patients (16) HCP (45)	Not reported (Discussing South Asian individuals)	End stage kidney disease	Hospitals	Action research methodology (including interview and focus groups) to explore issues relating to patient recruitment for research	82.5%	M		Y
Foliaki 2020 New Zealand [72]	Patients (7) Carers (15) HCP (33)	Pacific (93.3%) Non-Pacific (6.7%)	Cancer PC specialists GP Nursing staff	Hospice	Semi-structured interviews to explore perspectives on barriers to palliative care among Pacific populations	80%	M		Y
Omondi 2017 Kenya [73]	Patients (216)	African (69.9%) Asian (22.2%) Caucasian (6%) Other (2%)	Cancer ESKD Heart failure COPD	Hospital	Retrospective chart review to explore the amount of patients with advanced directives and factors affecting completion	80.6%	M	Y	

^a^Ethnic group names in this table are as per the original paper definitions

^b^JBI (Joanna Briggs Institute) score based on appropriate critical appraisal tool – average of two analysts (JC, ST)

^c^BI score used for WoE A (70–80% low, 80–90% medium, 90% + high)

^d^WoE (Weigh of Evidence) Score representative of WoE D (i.e., overall WoE)

### Primary outcome measure

Fifteen papers were included in the primary outcome measure analysis [15, 44, 47, 49, 51, 53, 56, 57, 59, 62, 64-66, 68, 73]. Only one paper came from outside of the USA (Kenya [73]), and no papers were located from the UK nor wider European countries.

All fifteen papers included drew comparison between two or more ethnic groups. The operationalisation of ACP was variable, as illustrated in Table 4; a general distinction can be drawn between the measurement of informal discussions (including EOL care discussions, advanced care planning conversations) and the measurement of medico-legal outcomes (including completion of legal or formal advanced care plans, completion of DNACPR forms, advanced directives, living wills, medical power of attorney and healthcare proxies). All papers defined ACP discussions and ACP outcomes as distinct concepts; six papers measured both [15, 44, 47, 51, 65].

**Table 4:** Synthesis of output for Research Question 1

First Author Year *Country*	ACP Data Collection Tool	Operationalisation of ACP	Ethnic Groups	Effect Measure	WoE^a^
Eneanya 2016 [44] *USA – Boston, Massachusetts*	Patient self-report *(structured interview)*	EOL care discussions Healthcare proxy DNR order	Black and African American (41.4%) White (58.6%)	Significantly more Black and African American patients had not completed a healthcare proxy form or DNR order compared to White patients *(51 vs. 30%; p* = *.01 and 78 vs. 62%; p* = *.04 respectively)* There were no significant ethnic difference in rates of EOL communication with healthcare professionals; 77% of all patients reported never having a prior EOL discussion with any healthcare provider. Significantly more Black patients reported not having discussions about EOL preferences with their family members or friends compared to White patients (54 vs. 27%, *p* = 0.01; Table 1); this persisted after adjusting for age, education, income, study site, CCI score and hospice knowledge (adjusted OR 2.70, 95% CI 1.08–6.76; Table 3)	H
Mack 2010 [47] *USA – multiple locations*	Patient self-report *(structured interview)*	EOL discussions DNR orders	Black and African American (21.4%) White (78.6%)	White patients were significantly more likely to have DNR orders than Black and African American patients *(50.4% vs 30.9%, P* = *.005)* There was no significant difference in rates of EOL discussions between Black and African American and White patients *(35.3% and 38.4%, respectively, P* = *.65)*	H
Pettigrew 2020 [49] *USA – multiple locations*	Patient self-report *(‘Care planning for individuals with dementia’ survey)*	Living will Power of attorney	Carer: White (87.2%) Black and African American (9.8%) Patient: White (88.2%) Black and African American (10.2%)	Amongst patients with dementia, significantly fewer Black and African American patients had “legal ACP” compared with White patients *(89% vs. 73%; OR* = *0.32, 95% CI (0.15, 0.71), p* = *.005)* However, there were no significant differences between Black and African American and White patients when legal and informal ACP were considered together *(95% vs. 88%; OR* = *0.37, 95% CI (0.13, 1.11), p* = *.08)* or when considering informal ACP* (80% vs. 70%; OR* = *0.57, 95% CI (0.28, 1.17), p* = *.13)*	H
Shen 2016 [51] *USA – New York*	Patient self-report *(structured interview)*	EOL care discussions DNR order	Hispanic (52.1%) White (47.9%)	The relative odds of signing a DNR order were significantly lower for Latino patients than White patients (*AOR* = *0.37, p* = *.049).* Latino patients who did not have EOL discussions were the least likely to complete DNR orders Based on multiple logistic regression models, the best fitting model (based on minimum AIC) included the main and interactive effects of Latino ethnicity and EOL discussions No significant difference in rate of EOL conversations between Latino and White patients (*34% vs. 42%, p* = *.349)*	H
Burgio 2016 [53] *USA – Birmingham, Alabama*	Deceased patient’s medical records	DNR order Advanced directive	Black and African American (34.5%) White (65.3%)	Black and African Americans were less likely than White patients to have a DNR order *(odds ratio* = *0.67 (0.55,0.84) p* = *0.004),* or an advanced directive *(odds ratio* = *0.71 (0.54,0.93) p* = *0.023).* In the multi-variable analyses controlling for other variables thought to be potentially related to the end points, racial differences remained significant	M
Garrido 2014 [56] *USA – Boston, Massachusetts*	Patient self-report *(structured interview)*	DNR order	White (72.1%) Black and African American (15.3%) Hispanic (12.5%)	Significantly more White patients *(45%)* reported having a DNR order than Black and African American or Hispanic patients *(25% and 20% respectively: p* < *.001)*	M
Grill 2021 [57] *USA – Washington*	Patient self-report *(The Lyon Family Centered ACP Survey-Patient Version Revised)*	Advanced directive Healthcare Power of Attorney	African American (86.1%) Non-African-American (11.2%)	36% of non-African Americans had completed advanced directives, compared to 12% of African Americans. 10 (40%) non-African Americans had written down thoughts about future care plans, in contrast with 58 (30.2%) African Americans	M
Kirtane 2018 [59] *USA – Washington*	Electronic health records and death certificates	Living will Healthcare power of attorney	White (84.2%) Black and African American (4.8%) Asian (6.4%) Pacific (0.5%) Hispanic (1.7%) Minority other (2.3%)	Non-White ethnicity was associated with a lower rate of advance care planning overall *(odds ratio* = *0.60 (0.43 – 0.82), p* < *.01),* and advanced care planning more than 30 days before death *(odds ratio* = *0.59 (0.42 – 0.83), p* < *.01).* However, rates of advance care planning within the last 30 days of life were roughly equal for the two racial groups	M
Phipps 2003 [62] *USA—Philadelphia*	Patient self-report *(structured interview)*	Advanced directive Healthcare power of attorney	Patients: African American (55.8%) White (44.2%)	White patients were significantly more likely to have a power of attorney *(34% v 8%, P* < = *.01)* and advanced directive *(41% v 11%, P* = *.004)* than African American patients	M
Sharma 2011 [64] *USA – Chicago, Illinois*	Medical chart review	DNR order Advanced directive Power of attorney	White (63%) Black (32%) Other (5%)	Black and African American patients were significantly more likely to have a power of attorney *(OR* = *0.41 (0.15 – 1.14), p* = *.01)* **No significant differences were found between Black and African American and White patients for DNR order *(OR* = *1.21 (0.68, 2.16) p* = *.51*) or advanced directive** *(OR* = *0.76 (0.36 – 1.58), p* = *0.46)* Compared to White patients, Black patients had higher odds of having a discussion about hospice (AOR 2.11; 95% CI 1.18–3.76) and being referred to hospice (AOR 2.18; 95% CI 1.21–3.93)	M
Smith 2007 [65] *USA – Boston, Massachusetts*	Patient self-report (*survey)*	Living will Health care proxy EOL discussions	Black and African American (14.3%) White (85.7%)	Significantly fewer Black and African American patients had at least 1 type of ACP than White patients (*47% vs. 79.5%, p* < *.001) (adjusted relative risk [aRR]* = *0.66, 95%CI* = *0.52–0.84)* Black and African American patients were significantly less likely than White patients to have a living will (*14.9% vs. 54.9%, p* < *.001),* or a health care proxy (*22.1% vs. 51.3%, p* < *.001)*	M
Smith 2008 [15] *USA – Boston, Massachusetts*	Patient self-report (*structured interview)*	Living will DNR order EOL discussions	White (69.4%) Black (16.7%) Hispanic (13.8%)	29% of patients discussed plans for EOL care with their physician, which did not differ by race/ethnicity. However Black and African American patients were 41% less likely, and Hispanic patients 40% less likely than White patients to have an ACP *(aRR, black vs. white patients, 0.59 [95% CI, 0.46 to 0.77]; Hispanic vs. white patients, 0.60 [95% CI, 0.45 to 0.82])*	M
Taylor 2015 [66] *USA – Houston, Texas*	Medical chart review	Living will Medical power of attorney	White (60%) Black and African American (16%) Asian (4%) Hispanic (20%)	Non-White patients were significantly less likely to have formal ACP documentation *(24% vs. 76%, p* = *.009)*. This remained even if they were enrolled in hospice *(12% vs. 31%, p* = *.007).* 67% of non-white patients did not have a medical power of attorney or a living will, compared to 24% of white patients; once enrolled in hospice this disparity corrected to 31% of non-white patients compared with 12% of white patients	M
Zaide 2012 [68] *USA – New York*	Review of medical charts	Advanced directive	White (47%) Black and African American (35%) Asian (11%) Hispanic (7%)	Black and African American patients *(p* = *.0005*) and Hispanic patients *(p* = *.0042)* were significantly less likely than White patients to have an advanced directive pre-palliative care consultation (PCC), but not post-PCC. Hispanics differed from whites in the overall rate of AD completion, irrespective of the timing (14.8% completed pre-PCC, 11.1% completed post-PCC, 74.1% never completed an AD – versus whites where 25.7% completed pre-PCC, 33.7% post-PCC, and 40.6% never)	M
Omondi 2017 [73] *Kenya – Nairobi*	Review of medical records Self-report (survey)	Advanced directive	African (69.9%) Asian (22.2%) Caucasian (6%) Other (2%)	41.2% of terminally ill patients had completed AD. **There was no statistically significant difference by ethnicity in advanced directive completion rate** *(p* = *.377)*	M

^a^Gough’s Weight of Evidence (WoE)

All fifteen papers used legal measures of ACP: Six papers measured DNACPR completion [15, 44, 47, 51, 56, 57], five measured advanced directive completion [53, 57, 62, 64, 68], and five measured living wills [15, 49, 59, 65, 66]. Thirteen papers found significant differences between ethnic groups [15, 44, 47, 49, 51, 53, 56, 57, 59, 62, 65, 66, 68]. Twelve of the thirteen papers found higher rates of legal ACP completion for White patients compared with patients from other ethnic groups (41,44,46,50,54,55,57,60,64,65,66,68]. One paper identifying ethnic differences in rates of ACP outcome found no significant differences between White patients compared with Black and African American patients in DNACPR or advanced directive completion but found that Black and African American patients were more likely to have a power of attorney [64]. Another paper carried out in Kenya found no statistically significant differences by ethnicity in advanced directive completion [73].

Of the thirteen papers finding statistically significant ethnic differences in the presence of legal ACP, four were rated High Weight of Evidence [44, 47, 49, 51]. The remaining nine were all rated Medium Weight of Evidence [15, 53, 56, 57, 59, 62, 65, 66, 68]. The two studies that reported no significant differences were also rated Medium Weight of Evidence [64, 73].

Five studies operationalised ACP in terms of informal discussion or end of life conversations [15, 44, 47, 51, 65]. None of these papers identified ethnic differences in rates of patients receiving ACP discussions with their healthcare providers, despite all five indicating statistically significant differences in legal ACP measures [15, 44, 47, 51, 65]. Of these five studies, three were rated High Weight of Evidence, and two Medium.

### Secondary outcome measures

The secondary outcome measures investigated ethnicity-related patient and clinician factors affecting presence of ACP in patients’ healthcare record. Nineteen papers addressed these outcomes and were included in the analysis [41-43, 45, 46, 48, 50-52, 54, 55, 58, 60, 61, 63, 67, 69-72]. The majority (eleven) of these papers were from the USA (39,40,45,47,49,51,56,59,61,63,67], with six based in Asian countries (42,43,52,58,69,70]; two in New Zealand (53,72); and a single UK-based study [71]. Individual studies varied widely in the ethnically diverse groups they assessed.

### Patient factors affecting ACP

Thirteen of the nineteen papers discussed patient-based factors affecting ACP presence; four key themes were identified: lack of awareness and understanding; financial constraints; faith and religion; and family involvement.

#### Lack of awareness and understanding

One frequently cited barrier to ACP was a general lack of awareness and understanding surrounding ACP [32, 41, 42, 45, 55, 58, 60, 63]; this was evident across studies of varied ethnically diverse groups, including Pacific patients within New Zealand, Indigenous patients living in Taiwan, Chinese and Malay patients in Singapore, and African American individuals [45, 60, 63, 72]. Participants stated they had very limited understanding surrounding the aims, options and procedure for ACP [72]. Patients also expressed confusion around the legalities of ACP, including around the role and authority of each participant in the decision-making process, and whether an individual could change their mind at a later date [60]. Of the eight studies which reported a lack of awareness as a barrier to ACP, four explicitly stated this; all four of these involved participants with cancer [45, 60, 63, 72].

*“All patients were predominantly unaware of concepts related to ACP, and none of the cases, nor their family caregivers, had prior experience making life-sustaining treatment (LST) decisions themselves.”* [45].

There are multiple intersectional factors which impact upon a person’s awareness of ACP. The evidence suggests that a lack of previous experience with, and exposure to, death and dying may contribute to an individual’s lack of awareness surrounding ACP. Beltran (2022) in an American study of Latino patients and relatives found that minimal exposure to other relatives’ ageing and dying limits the opportunity to learn about ACP and facilitate conversations about one’s own preferences [42]. Highlighted reasons for this lack of exposure included immigration to another country resulting in elder relatives living far away, and lack of funds to visit elder relatives who were reaching end of life. Where individuals did have previous experience caring for an unwell relative, they were often more agreeable to conversations surrounding their own ACP [46].

An individual’s health literacy and education levels may also impact their understanding of ACP. Patients perceived “*limitations in their ability to seek out and understand health information and services”* [42]. Li (2021) found that amongst people from Taiwanese Indigenous tribes, patients with lower levels of formal education retained a lower readiness to engage in ACP even after exposure and awareness promotion [45].

#### Financial constraints

Financial necessity influenced end-of-life consultations particularly in countries where ACP consultations were not covered by insurance, nor subsidised by the healthcare system [46]. In the UK, the impact of financial constraints is more ambiguous due to a largely accessible and freely available National Health Service. However, PPI contributors emphasised that, financial constraints do play a part, particularly for people who have No Recourse to Public Funds (NRPF) [74].

#### Faith and religion

Of the thirteen papers discussing patient-based factors, ten discussed the importance of faith and religion for ACP [41, 45, 48, 50, 51, 54, 55, 58, 63, 69]. The belief that ‘one’s journey is in God’s hands’ heavily impacted opinions on and willingness to engage in ACP and was seen across papers with participants from a number of ethnically diverse groups (Latino [54]; Indigenous to Taiwan [45]; Asian Americans [48]). For some Latino patients, signing an advanced directive was seen as not having faith in God, as God is perceived as the determinant of death [54]: engaging in ACP is deemed futile and disrespectful of their pre-ordained fate. A sample of Indigenous patients with late-stage cancers expressed that life-sustaining treatment was unnatural, and would contest God’s authority [45].

For some patients, faith that their health-trajectory was not in their control, allowed for optimism and positivity despite difficult circumstances. In an American study of Latino cancer patients, patients described remaining optimistic as they placed their faith in the ability of God to guide their lives towards a positive outcome [51]. Similarly, some Chinese patients spoke about “a*ccepting the inevitability of death and staying positive”* [55]. Jung-Hwa (2021) highlighted “*an old saying in Korea… “Even if I am rolling in dog poop, this life is better than the one in the afterlife.”* referring to th*e* preference to focus on the positives, rather than preparing for the worst [69, 71]*.*

#### Family involvement and looking after your own

Nine of the thirteen papers identified family as a factor affecting discussions around ACP (45,47,51,52,53,56,59,63,69,72). For some patients, this meant entrusting all end-of-life decisions to family members [48, 51, 55, 69]. For others, initiating ACP conversations caused apprehension for fear of causing distress to loved ones [54] and disturbance to daily lives [55].

In some cultures, caring for loved ones was considered the family’s responsibility – a “fundamental family obligation” [72]—particularly following the diagnosis of advanced disease. In this context, it was reported that ACP was perceived by some to represent evasion of this responsibility by avoiding doing everything they could for their relative, particularly for Pacific and Asian families [41]. Allowing a relative to be admitted to a hospice in this context was a source of shame and embarrassment [72].

### Clinician factors affecting ACP

Of the nineteen papers addressing the secondary outcome measure, eight examined clinician-based factors affecting ACP presence (40,43,45,47,49,52,67,71]. Qualitative analysis identified three key themes: poor clinician confidence around cultural values and ideals; exacerbation of institutional constraints; and pre-conceived ideas of patients’ wishes.

#### Clinician competence and confidence

Four papers indicated that clinician confidence in initiating and delivering ACP conversations was key [43, 48, 52, 71]. Uncertainty around prognosis and the timing of ACP conversations was a source of hesitation; particularly for clinicians with perceived poor understanding of diverse cultural values and ideals around death and dying, who felt ill-equipped to sensitively deliver ACP [71]. Uncertainty also influenced concern around potentially causing distress to patients via inappropriately addressing ACP [52, 71].

*“The doctors felt that their ignorance about the diverse cultural values around death and dying was the third biggest barrier to effective EOL conversations…this led to doctors committing cultural faux pas by discussing taboo topics which inadvertently offended the patient/family and undermined the therapeutic relationship”* [48].

For some clinicians, the interaction of their own ethnicity with their patient’s ethnicity, had an impact upon their approach to conversations about ACP. An American study comparing White, African American and Hispanic clinicians found that some African American physicians stated they would “*approach EOL discussions with patients of their own race differently than with race-discordant patients”* [43], though the content would remain the same. In contrast, White doctors in the same study considered concordance of ethnicity to be less relevant and reported that end of life issues “*are colour blind and apply to everyone regardless of race”* [43].

#### Insufficient institutional resources

Seven of the eight papers identified exacerbation of resource limitations as a potential factor impacting ACP presence [43, 46, 50, 52, 55, 67, 71].

One key resource was time: in an American study of healthcare professionals, 91% of clinicians reported time constraints as a barrier for completing ACP conversations with patients, particularly where “*cultural factors that influenced the perception of palliative care were a barrier to their practice*” [67]. In another study examining the feasibility of a culturally sensitive ACP intervention, “a*dvance care planning was viewed as increasing staff workload in an already busy routine and additional resource was required to embed the intervention into clinical practice*” [46]. Time constraints were compounded for patients with language barriers, where further time was required to source an interpreter and then communicate through them. Further difficulties here included accessing interpreters when required, and accessibility of ACP resources both in other languages and in more accessible versions for patients with limited health literacy [52].

Many clinicians believed that wider resource constraints could be somewhat eased by sufficient support from their workplace and the healthcare system in question. For example, some reported that a clear protocol for the delivery and recording of ACP conversations improved clarity around the process [43] and could increase involvement of other healthcare professionals (for example, social workers), increasing dissemination and reducing the burden of ACP on clinicians. The role of workplace support was also described in the context of its lack thereof; some clinicians felt they received *“insufficient recognition by colleagues of the importance of palliative care”* [67].

#### Clinician pre-conceptions

A barrier to ACP conversations identified in the literature was clinicians' unconscious pre-conceived ideas and stereotypes about patients’ thoughts, wishes, and preferences [43, 48, 52]. Entering an ACP conversation with these preconceptions could limit patients’ opportunity to properly consider their preferences [43, 52]. In some cases, *“Clinicians’ preconceived views about discussing ACP with structurally marginalized patients resulted in their avoiding ACP altogether”* due to the assumption the patient would not engage [52].

Perceived trust was also relevant here: physicians often felt as though patients from diverse and minoritised communities did not trust them or the healthcare system and conceded that patients would therefore not engage in ACP [43, 48]. For some clinicians, this belief was borne from awareness of patients’ past experiences [43]. Interestingly, some clinicians reported beliefs of patient distrust, yet reported that they were not perceived to be distrustful themselves [48].

## Discussion

To our knowledge, this is the first review to examine ACP presence and the factors affecting this across all ethnicities and all disease groups, globally.

### Evidence from the primary outcome measure analysis

Twelve papers found that presence of at least one legal measure of ACP (i.e., DNACPR, power of attorney, advanced directive or others) was statistically significantly more common amongst White patients than amongst patients from minoritised ethnic groups [15, 44, 47, 49, 53, 56, 59, 62, 65, 66, 68]. One paper found no statistically significant differences by ethnicity [73]. This paper was set in Kenya, where the majority ethnicity was people from African groups (69.9%), with other minorities including people from White groups comprising 6%. Unlike most of the papers included in this review, this paper is set outside of the Western healthcare system. This may indicate that the power imbalances between ethnic groups present within Western systems of healthcare, may not be present, or may look different, in other area of the World. However, there is not enough data to draw conclusions here. This may be a topic for further research exploration.

All five papers that operationalised ACP in terms of informal discussion or conversation about end of life found no statistically significant ethnic differences in this measure [15, 44, 47, 51, 65], despite all five papers reporting statistically significant differences in legal ACP measures. Whether all patients were offered legal ACP as part of these informal discussions at the same rate is a question for further research.

#### Patient-based factors affecting ACP presence

Four key themes pertaining to patient-related factors affecting presence of ACP were identified: lack of awareness and understanding; financial constraints; faith and religion; family involvement and dynamics.

The evidence showed that lack of awareness and understanding of ACP by patients was a barrier. This is consistent with a review by Hong et al. (2018), which set out that lack of knowledge is a principal barrier to ACP in minoritised ethnic groups [33]. This current finding could be considered in relation to intersectionality of ethnicity and health literacy: intersectionality is an acknowledgement of the ways that multiple forms of inequity can compound themselves, creating obstacles [75]. Intersectional factors of health inequity and health literacy have been recently evidenced by Suurmond (2021), who recommended introducing a curriculum point around health literacy within palliative care education to improve awareness of the prevalence and challenges of poor health literacy within some minoritised ethnic groups [76]. This would promote consideration of this when discussing preferences with patients. Okoro et al. (2022) recently discussed this with relation to intersectional invisibility: “possessing multiple subordinate-group identities renders a person “invisible”” [77, 78]. If patients from minoritised ethnic groups have poor health literacy, and consequently feel invisible during conversations with clinicians, this will likely result in undesired care pathways for the end of life.

Faith and religion were found to frequently impact the acceptability of ACP. For some participants, the end of life was pre-ordained by God; planning for the end of life was perceived to undermine God’s authority, or to be unnecessary as their end of life had already been planned by a higher being. Consequently, some faith-centred ethnic groups are choosing not to engage with ACP. Religion was not specifically measured in this study, though it should be noted that existing studies often consider ethnicity and religion as intertwined [32]. This makes it difficult to truly assess the impact of religion on ACP in ethnically diverse groups; further research is needed to understand this relationship.

The role of a patient’s family was found to vary within and across ethnicities: some individuals preferred to make decisions alone and avoid ‘burden’ to their family, while others placed importance on collaborative familial decision-making. The position of individuals and their families regarding familial or self-expression of end of life preferences may impact the perceived relevance and importance of ACP, in turn impacting engagement with ACP conversations and documentation.

#### Clinician-based factors affecting ACP

Confidence and competence were important factors for clinicians undertaking conversations about ACP. Papers often reported clinicians having poor understanding around cultural values and ideals; related to this was poor cultural competence where clinicians held preconceptions that influenced rates of initiation of conversations about ACP [79].

Previous work suggests that White doctors are less likely than doctors from minoritised ethnic groups to believe that ethnic l inequality in healthcare exists [80, 81]. Similarly, here, some White doctors perceived end of life issues as “colour blind and apply to everyone regardless of race” [43]. This implicit tendency to see the White population as a normative standard [82], while underplaying the severity of ethnic inequalities in healthcare, risks further exclusion of minoritised ethnic groups from engaging in ACP.

Clinicians were generally willing to discuss ACP with patients but lacked the confidence and cultural understanding to do so [83]. The evidence from this review indicates a training need for clinicians around culture, ethnicity and conversations about ACP. One potential model for facilitating these conversations is the Cultural Humility model [84], which has three tenets: lifelong learning and self-reflection; mitigating power imbalances; and institutional accountability. It does not view cultural differences as reified ‘facts’, but rather places the patient as ‘expert’, and requires that cultural knowledge is utilised as part of an ongoing conversation between doctor and patient. Further, the “Platinum Rule” of “doing unto patients as they would want done unto themselves” could provide an important contextual guide for clinicians who are new to, or lack confidence in, inter-ethnic healthcare [26]. Training in using the cultural humility model of communication for ACP could improve clinician confidence whilst simultaneously addressing patient related factors.

#### Patient & public involvement

Contributions from Patient and Public Involvement representatives informed the analysis of the secondary outcome measure. They largely agreed with the shaping of the six key themes representing patient and clinician factors affecting ACP presence. It is important to note that contributors were shocked to read findings around clinicians’ pre-conceived ideas of patients wishes; they explained that it was unnerving that this stereotyping occurs, particularly when they have not knowingly been subject to it themselves.

PPI contributions emphasised the importance of early ACP intervention. Timing was highlighted as a challenging clinician-related factor, whereby clinicians wanted to introduce ACP at a prognostically-appropriate juncture; in contrast, PPI representatives suggested introducing ACP much earlier, to prevent it from occurring too late to be a viable and effective option for patients. A South Asian representative highlighted how ACP should encompass broader questions, including questions around wills and other legal issues; they described poor understanding of the need for a will in their community due to the misplaced assumption that Sharia Law would be in place. Exposure to death and dying was highlighted as an important factor in awareness of ACP; a White Jewish representative shared their emotive experiences around the deaths of two close friends, and how this led them to develop and discuss openly with their family their own wishes and plans for death.

## Limitations

### Heterogeneity of ethnicity data & limitations in individual reports of ethnicity

The collection of data regarding ethnicity is often heterogeneous and incomplete. Research literature often does not specify the methodology used to assign participants to specific ethnic categories (72% of papers had an absence of explanation [85]). Moreover, electronic medical records ethnicity often do not align with participants’ self-reported ethnicity [86]. Some individual studies included in this review used somewhat biased and reductive language (for example: “ethnic minorities” or “White vs non-White”) [9]. This inconsistency in ethnicity data from individual studies forced some assumptions and groupings on our part (see Table 2 – groupings for summary analysis). We attempted to overcome this by using previously peer reviewed groupings as a guide [87], and consulting PPI representatives on the appropriateness of the groupings. To improve this in future research, ethnicity data collected within a research capacity must be detailed, individualised and represented within the social context.

It should also be highlighted that this review was limited to differences based on ethnicity. As discussed above, ethnicity and religion are often considered intertwined; these facets also relate to broader individuality. This review aims to highlight learning points and areas where culturally competent ACP can be improved; it is important to note that a patient-centred approach is key.

### *Researcher *positionality

As noted by Manohar et al. (2017), a researcher’s background, experiences and positionality will undoubtedly impact their perspective and interpretation of the data, even if this is implicit [88]; it can be argued that no knowledge or research can be completely neutral [89]. In the current study, three members of the research team are White British. Of the patient and Public Involvement co-authors, one is South Asian and another identifies as Jewish Other White ethnicity. All five authors are female.

### Operationalisation of ACP

Although this review demonstrated no statistically significant differences between ethnicities in informal ACP conversations, “presence of conversation” is quite a crude measure. Furthermore, the current review did not look at content of conversation nor how successful it was deemed by the clinician subsequently, nor at the effect on the patient subsequently.

### *English *language

A potential weakness of this review is the exclusion of any non-English language papers. This was a decision of necessity based on the authors’ skillset. It should also be noted that the majority of included papers were from the USA (in part, a reflection of the lack of data on this question from the UK); thus the current dataset may not be representative of ACP universally. Expansion of the eligibility criteria to include non-English language papers may have identified more high quality, rich datasets that are important to this review.

## Conclusion

The current review investigated potential differences in the documented presence of ACP in patients’ care records by ethnicity. Assessment of the primary outcome measure found statistically significant differences in the documented presence of legal ACP measures by ethnicity in countries where majority is White; people from White groups were more likely to have legally documented ACP in their health record compared to people from other groups. However, no statistically significant differences were found in presence of discussions around informal ACP.

From the clinicians’ perspective, factors which were considered barriers to ACP included resource limitations (particularly of time) and a lack of confidence in holding culturally sensitive discussions about ACP. From the patients’ perspective, factors affecting ACP included a lack of understanding or awareness of ACP; resource limitations (particularly financial); and factors such as religion, faith, and family. Further research is needed to understand how to deliver individualised, culturally sensitive ACP conversations to minoritised ethnic groups, to provide equitable opportunity to make informed decisions.

### Recommendations

In order to move towards more equitable ACP, we propose the following recommendations for research, policy and practice:

- Increased research on intersectionality in advance care planning internationally, particularly within the UK: Research identified within this review was mainly based within the USA, meaning it may have limited generalisability across other countries, particularly due to differences in sociocultural contexts and healthcare systems. An increase in research (both original investigations of different ethnic groups and replications of USA-based studies) in a variety of understudied regions would give insight into the transferability of findings and improve understanding of ACP values in relation to different cultural values.
- Greater policy and healthcare engagement to allow earlier introduction of culturally safe and sensitive ACP to all patients: There was a lack of awareness and understanding of ACP as an ‘unfamiliar’ idea amongst participants of incorporated studies. Reinforced by feedback from PPI contributions, normalising ACP conversations through early culturally-sensitive introduction may promote equitable access and information about ACP, allowing people time to engage with conversations, if they choose to do so, and recognising where, when and for whom it may not be appropriate.
- Greater patient-centredness using a cultural humility model: This study identified a lack of understanding of different cultural ideals and values as a clinician-based barrier to discussion about ACP. There is potential for more education and training around patient-centred ACP; the cultural humility model and Platinum Rule could be helpful here.

## Supplementary Information

**Additional file 1:** **Appendix Table 1.** PRISMA 2020 item checklist.

## Data Availability

Original EndNote libraries containing search output, and any other materials, can be provided on reasonable request to corresponding author. Please contact the lead author Jodie Crooks via email (Jodie.Crooks@mariecurie.org.uk).

## Abbreviations

AMED: Allied and Complementary Medicine Database
CINAHL: Cumulative Index to Nursing and Allied Health Literature
EMBASE: Excerpta Medica database
JBI: Joanna Briggs Institute
PPI: Public and Patient Involvement
PRISMA: Preferred Reporting Items for Systematic reviews and Meta-Analyses
UK: United Kingdom
USA: United States of America
WoE: Weight of Evidence
